# Computational methods for prediction of in vitro effects of new chemical structures

**DOI:** 10.1186/s13321-016-0162-2

**Published:** 2016-09-29

**Authors:** Priyanka Banerjee, Vishal B. Siramshetty, Malgorzata N. Drwal, Robert Preissner

**Affiliations:** 1 Structural Bioinformatics Group, Institute for Physiology, Charité – University Medicine Berlin, Berlin, Germany; 2 Structural Bioinformatics Group, Experimental and Clinical Research Center (ECRC), Charité – University Medicine Berlin, Berlin, Germany; 3 Graduate School of Computational Systems Biology, Humboldt University of Berlin, Berlin, Germany; 4 BB3R – Berlin Brandenburg 3R Graduate School, Free University of Berlin, Berlin, Germany; 5 Laboratoire d’innovation thérapeutique, Université de Strasbourg, Illkirch, France

**Keywords:** Similarity searching, Machine learning, Toxicity prediction, Tox21 challenge, Molecular fingerprints

## Abstract

**Background:**

With a constant increase in the number of new chemicals synthesized every year, it becomes important to employ the most reliable and fast in silico screening methods to predict their safety and activity profiles. In recent years, in silico prediction methods received great attention in an attempt to reduce animal experiments for the evaluation of various toxicological endpoints, complementing the theme of replace, reduce and refine. Various computational approaches have been proposed for the prediction of compound toxicity ranging from quantitative structure activity relationship modeling to molecular similarity-based methods and machine learning. Within the “Toxicology in the 21st Century” screening initiative, a crowd-sourcing platform was established for the development and validation of computational models to predict the interference of chemical compounds with nuclear receptor and stress response pathways based on a training set containing more than 10,000 compounds tested in high-throughput screening assays.

**Results:**

Here, we present the results of various molecular similarity-based and machine-learning based methods over an independent evaluation set containing 647 compounds as provided by the Tox21 Data Challenge 2014. It was observed that the Random Forest approach based on MACCS molecular fingerprints and a subset of 13 molecular descriptors selected based on statistical and literature analysis performed best in terms of the area under the receiver operating characteristic curve values. Further, we compared the individual and combined performance of different methods. In retrospect, we also discuss the reasons behind the superior performance of an ensemble approach, combining a similarity search method with the Random Forest algorithm, compared to individual methods while explaining the intrinsic limitations of the latter.

**Conclusions:**

Our results suggest that, although prediction methods were optimized individually for each modelled target, an ensemble of similarity and machine-learning approaches provides promising performance indicating its broad applicability in toxicity prediction.

**Electronic supplementary material:**

The online version of this article (doi:10.1186/s13321-016-0162-2) contains supplementary material, which is available to authorized users.

## Background

The number of new chemical entities launched every year has been steadily increasing over the last decades irrespective of the number of successful drug approvals. High attrition rates in late stage of clinical trials are one of the most important reasons for the significantly low number of new drug approvals. The lack of efficacy and unfavourable safety profiles contribute the most to high attrition rates. Reviews indicate an increasing number of ‘me-too’ drugs that hardly provide an advantage over the existing therapeutics [1]. In an attempt to evaluate different drug discovery strategies, it was observed that the percentage of newly approved small molecule drugs with a novel molecular mechanism of action is less than 20 % of the total approvals during the study duration considered [2]. Currently, the majority of drug candidates are aimed at cancer treatment and are therefore studied for activity at multiple, possibly novel biological targets, presenting a high probability of multiple unique toxicological profiles [3]. Therefore, it is essential to employ novel strategies that can predict the fate of the chemicals in early stages of development to overcome the failure rates and accelerate the development and approval of promising candidates. Predictive toxicology, more commonly known as in silico toxicology, plays a key role in the optimization of hits by parallel investigation of safety and activity, thereby permitting a more efficient drug development process [4]. Along with in vitro assays, predictive toxicology received, in recent times, great attention as a method to evaluate various toxicological endpoints and reduce animal experiments, complementing the theme of replace, reduce and refine (3Rs) [5]. Additional factors that motivate the development of toxicological prediction methods include considerable progress with legislations in both the European Union and North America and the need for the reduction of costs involved in experimental testing of an increasing number of chemicals, as well as advances in the understanding of the biology and chemistry of the active chemical compounds.

The early efforts for prediction of toxicity date back to the 1890s, as emphasized by the work of Richet [6], Meyer [7] and Overton [8] on the relationship between toxicity and solubility followed by their hypothesis that narcosis could be related to partitioning between water and oil phases. Since then, steady progress has been observed in predictive toxicology, highly complemented by advances in cheminformatics approaches such as quantitative structure–activity relationship (QSAR) modeling [9], physicochemical property and molecular descriptor based modeling [10, 11] and statistical methods [12]. Later, a number of commercial and open-source expert systems have been developed for the prediction of pharmacokinetic parameters including TOPKAT^®^ [13], ADMET Predictor™ [14], ADME-Tox Prediction [15], DEREK [16] and Toxicity Estimation Software Tools [17]. Machine learning methods have been widely used in the areas of bioactivity and ADMET (absorption, distribution, metabolism, excretion and toxicity) properties prediction [18–23]. It has been demonstrated that models built with machine learning methods which take into account high-dimensional descriptors are very successful and robust for external predictions [24, 25].

The US toxicology initiative, Toxicology in the 21st Century (Tox21), started in 2008, aims to develop fast and effective methods for large-scale assessment of toxicity in order to identify chemicals that could potentially target various biological pathways within the human body and lead to toxicity [26]. The objectives of this initiative, after the initial screening, are to prioritize chemicals for further investigation of toxic effects and progressively build toxicity models as well as develop assays that measure responses of human pathways towards these chemicals. As a part of the screening initiative, a library comprising more than 10,000 chemicals was screened in high-throughput assays against a panel of 12 different biological targets involved in two major groups of biochemical pathways: the nuclear receptor pathway and the stress response pathway. Further, during the Tox21 Data Challenge 2014 [27], the development of computational models which can predict the interference of these chemicals in the two groups of pathways was crowd-sourced to researchers across the globe. Our previous work [28] illustrates the usefulness of a combination of chemical similarity and machine-learning approaches in predicting the activity of the Tox21 dataset with high accuracy for a majority of the targets considered in the challenge [29]. In this study, we present and discuss various computational methods, ranging from molecular similarity to different machine-learning approaches and their intrinsic limitations by comparing them with the best prediction models from our previous work [28] that ranked top among the submissions to the challenge. In order to keep the comparison simple, we limit ourselves to a set of three targets: aryl hydrocarbon receptor (AhR), estrogen nuclear receptor alpha ligand-binding domain (ER-LBD) and heat shock protein beta-1 (HSE). We also emphasize on the factors that can be attributed to a mixed performance of these models via illustration of example compounds.

## Results

**Fig. 1:**
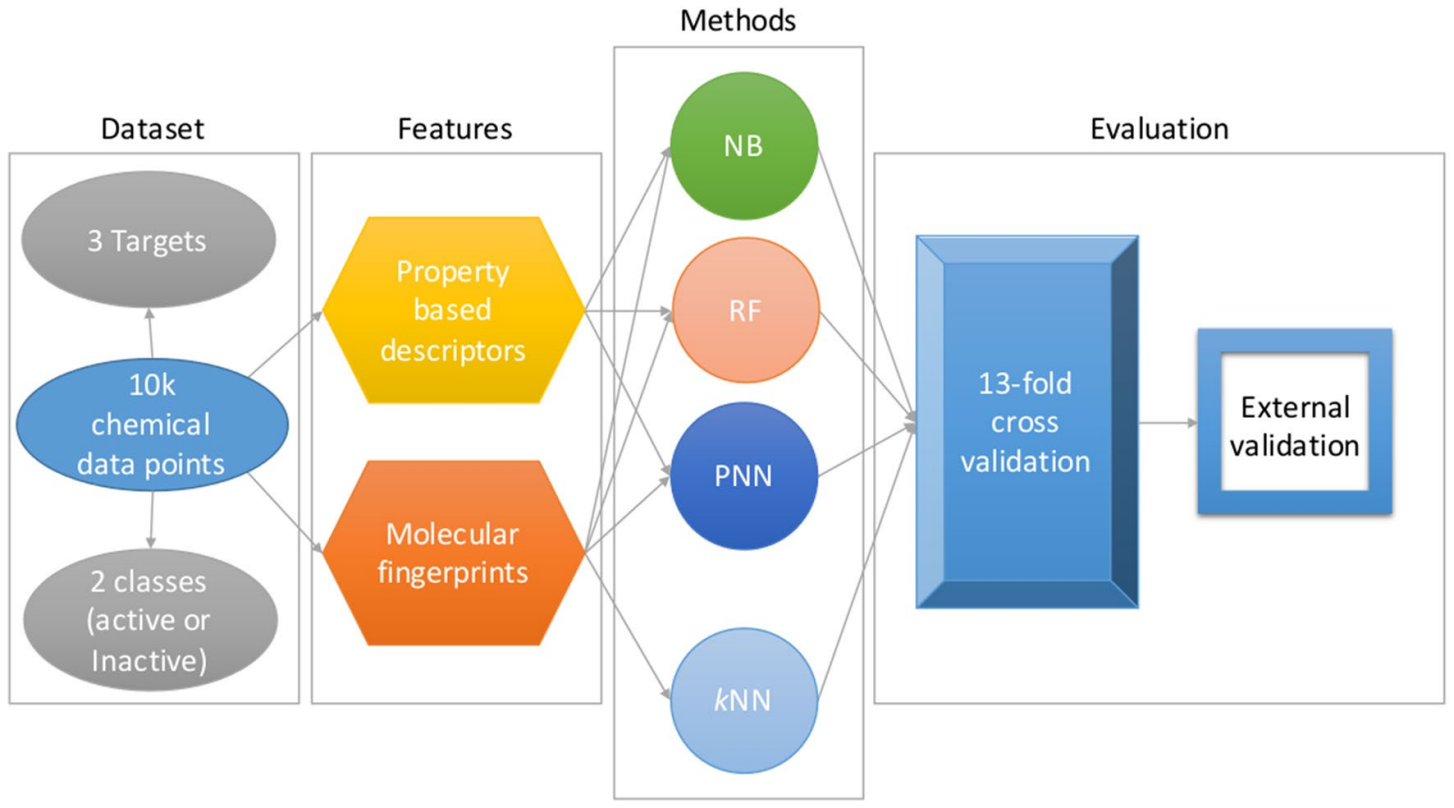
Workflow of the methodology involved in the classification process. Schematic representation of the methodology: data points, feature selection, model development (machine learning and similarity search methods) and validation, implemented in the study

We compared the performance of four different algorithms as well as four different molecular fingerprints for the prediction of the AhR, ER-LBD and HSE assays for the Tox21 10 K compound library (for more details, see Additional file 1: Tables S1, S2). In particular, similarity-weighted *k*-nearest neighbors (*k*NN) approaches as well as three types of machine learning algorithms (Fig. 1) were investigated, as described in detail in the Methods section. In order to evaluate the performance of different fingerprints used as a hybrid fingerprint in our previous work [28], we investigated MACCS [30], ECFP4 [31] and ToxPrint [32–34] fingerprints individually. While MACCS fingerprints are based on generic substructure keys, ToxPrint fingerprints encode generic substructures considering genotoxic carcinogen rules and structure-based thresholds relevant to toxicology. Extended connectivity fingerprints such as ECFP4 are based on the circular topology of molecules and have been designed for both similarity searching and structure–activity modeling. In addition, we chose to use ESTATE [35] fingerprints, to examine whether molecular fragments based on the electronic, topological and valence state indices of atom types can help in prediction of toxic activity. In addition to fingerprints alone, we also tested the concatenation of fingerprints with 13 selected molecular descriptors characterising the molecule’s topology and physicochemical properties (see “Methods” section and Supplementary Information). The performance of all models was investigated in cross-validation and external validatio. The best classifier for each target was selected based on the AUC values of the models generated.

### Similarity search based predictions

**Fig. 2:**
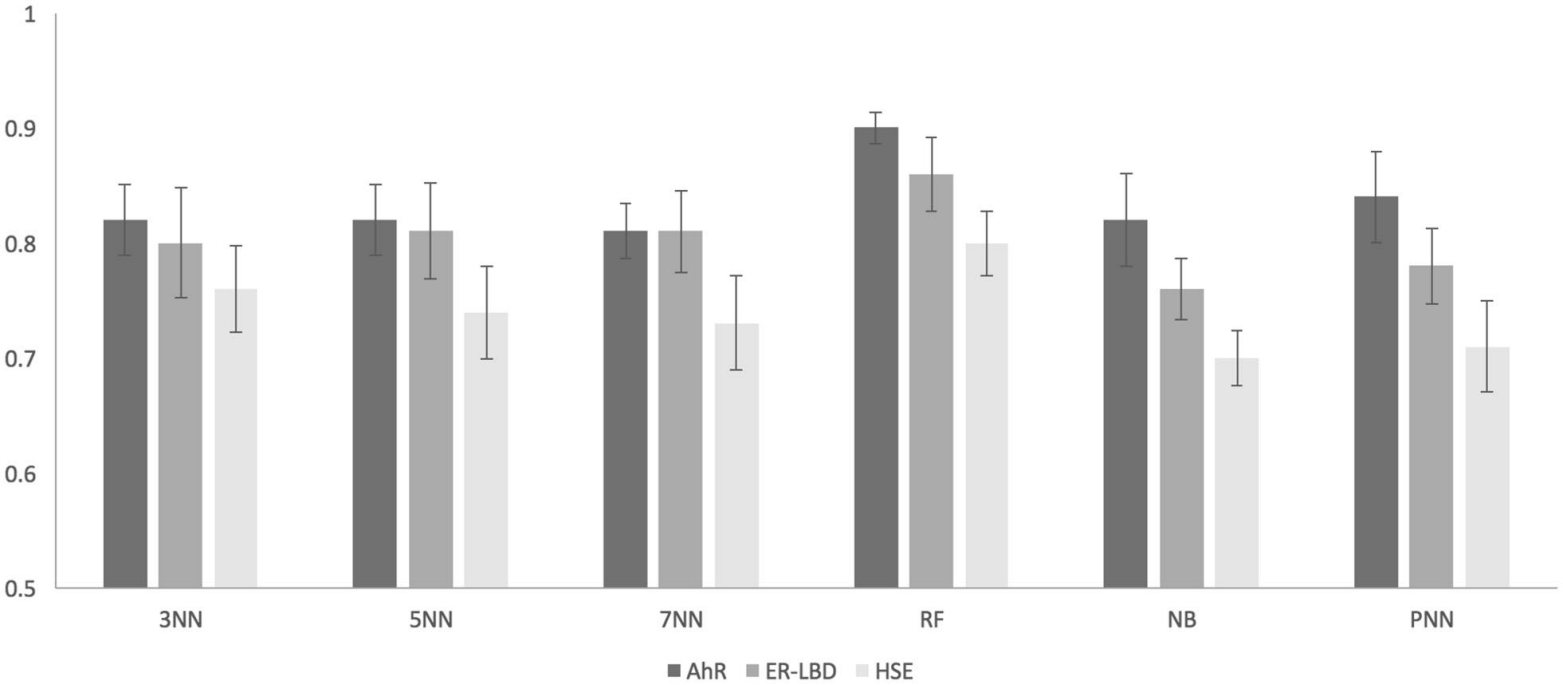
Cross-validation performance results of classifiers. Plot representing the 13-fold cross-validation results, in terms of AUC, for the three targets (AhR, ER-LBD and HSE) comparing different best performing models (3NN, 5NN, 7NN, RF, NB, and PNN) [28]

**Fig. 3:**
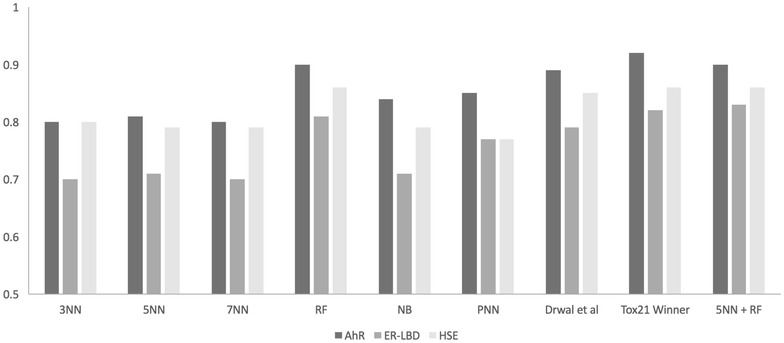
External validation performance results of classifiers. Plot representing the external validation results, in terms of AUC, for the three targets (AhR, ER-LBD and HSE) comparing different best performing models (3NN, 5NN, 7NN, RF, NB, PNN, Ensemble (5NN + RF)) with our previous work [28] and Tox21 challenge winners for respective targets

In the first step, we implemented a similarity-weighted *k*NN search with three different ‘*k*’ parameters (3, 5 and 7). It was noted that all three *k*NN approaches based on the MACCS fingerprint performed better than those based on ECFP4, ESTATE and ToxPrint fingerprints in cross-validation and external validation. The AUC values achieved with the best performing fingerprint for each target are presented in Fig. 2 (cross-validation with error bars) and Fig. 3 (external validation) and those for all other fingerprints are available in the Supplementary Information (Additional file 1: Tables S3, S4). With all the *k*NN models for AhR and HSE, ESTATE and ToxPrint fingerprints performed similarly to MACCS fingerprints followed by ECFP4 with the least performance. All models for ER-LBD showed the worst performance compared to the other two targets.

For AhR and ER-LBD, the *5*NN approach performed better than the *3*NN and *7*NN approaches. The *3*NN method, however, achieved clearly better performance for HSE. These observations were true for both cross-validation (Additional file 1: Table S5) and external validation (Additional file 1: Table S6) results.

Overall, the similarity-weighted *k*NN approaches showed target-dependent results with better performance on AhR (mean AUC = 0.81) and HSE (mean AUC = 0.8) compared to ER-LBD (mean AUC = 0.71) in both cross-validation and external validation.

### Machine learning predictions

Three different models, a Naïve Bayes (NB), random forest (RF) and probabilistic neural network (PNN) classifier (see “Methods” section for details) were developed. Additionally, we have tested support vector machine (SVM) models with both a linear and a polynomial kernel function. However, the performance was not consistent across different targets and descriptors, and was therefore not considered further. A small description as well as the results of SVM can be found in the Supplementary Information (Additional file 1: Tables S7 and S8).

In this study, almost all the classifiers reached prediction accuracies around 80 %. Since the data set used in this study is highly imbalanced (Additional file 1: Tables S1, S2), accuracy alone cannot reflect the performance of the models. We have further evaluated the models based on the ROC AUCs that represent more accurately the performance of the models.

Based on our analysis using cross-validation and external validation, RF models perform best for all the three targets and PNN models show the least performance (Additional file 1: Tables S3, S4). A comparison of different molecular fingerprints and their combination with the molecular property based descriptors for different models on cross-validation sets as well as external validation set have been provided in the Supplementary Information (Additional file 1: Tables S7, S8).

The RF based model for AhR showed a good performance with MACCS, ECFP4 and ToxPrint with an AUC value of above 0.88 on the cross-validation sets as well as the external validation set. However, the MACCS fingerprint individually and combined with molecular property-based descriptors obtained the highest AUC value of 0.90 and 0.91 (cross-validation) and an AUC of 0.90 and 0.87 (external set) (Figs. 2, 3). The combination of descriptors did not improve the external set performance in this case. Similarly, MACCS fingerprints scored highest with AUC values of 0.83 and 0.80 (cross-validation) and 0.81 and 0.86 (external set) for ER-LBD and HSE, respectively (Figs. 2, 3).

Furthermore, the NB based model with MACCS fingerprints in combination with molecular property-based descriptors and ToxPrint fingerprints performed comparatively good for AhR with an AUC value of 0.84 and 0.82 respectively. The performance for ER-LBD and HSE were relatively poor with an AUC value below 0.75 for both cross-validation sets and external set. The PNN classifier performed better for AhR, with an AUC value above 0.80 for almost all the descriptor combinations (Additional file 1: Tables S7, S8). These results could be explained by the lack of a balanced dataset which could have a negative impact on the performance of PNN and NB based models. On the other hand, it is observed that the RF algorithm performs well on imbalanced datasets.

To generalize, it is observed that MACCS fingerprints based on RF classifier, similarly to the similarity-weighted *k*NN approach, exhibit the best performance (Additional file 1: Tables S3, S4). An exception is the AhR assay, where in ToxPrint fingerprints performed equally well with an AUC value of 0.89 and 0.88 (Additional file 1: Tables S7, S8) for the external dataset and cross-validation sets respectively, when compared to the method reported in our previous work [28]. Since the training set as well as the number of active molecules available for AhR was relatively large when compared to ER-LBD and HSE, it reflects that the size of the training set as well as the ratio between active and inactive molecules is one of the factors contributing to its better performance (Additional file 1: Tables S1, S2).

### Comparison and combination of similarity and machine learning methods

In comparison to similarity search approaches, the RF based machine-learning models performed better for all three targets in external validation (Fig. 3). However, both approaches performed equally well in cross-validation. Assuming that the inferior performance of similarity-based approaches is due to the fact that the actives in the external set share little structural similarity with the actives in the training set, we combined our best performing similarity approach with the best performing RF model in order to improve the prediction. For each of the three targets, the scores from the *5*NN method and the RF model (*5*NN + RF), both based on MACCS fingerprints, were combined. It was observed that the performance improved for ER-LBD with an AUC value of 0.83 in external validation (Fig. 3) and 0.85 in cross-validation, using a minimum of the prediction scores from both models. However, the RF model remained the best performer for the targets AhR and HSE as no additional improvement was observed with the *5*NN + RF model.

### Analysis of chemical space based on RF and NB based models

In the next step, we evaluated the patterns associated with active chemical structures by analysing the compounds, which were correctly and incorrectly predicted by respective models in case of ER-LBD for the external set (Tables 1, 2). Since we achieved the best performance for ER-LBD using an ensemble method, it is of particular interest to investigate which chemical characteristics were correctly predicted by different methods and fingerprints (MACCS, ECFP4).

**Table 1:** Classification of actives and inactives in external set by different models for ER-LBD

ER-LBD	True positives/actives (out of 20)	True negatives/inactives (out of 580)	Cross-validation AUC	External set AUC
NB with ECFP4	9	500	0.76	0.71
NB with MACCS	8	468	0.73	0.69
RF with ECFP4	2	574	0.82	0.78
RF with MACCS	4	576	0.83	0.81
PNN with ECFP4	0	580	0.77	0.69
PNN with MACCS	0	580	0.78	0.69

**Table 2:**
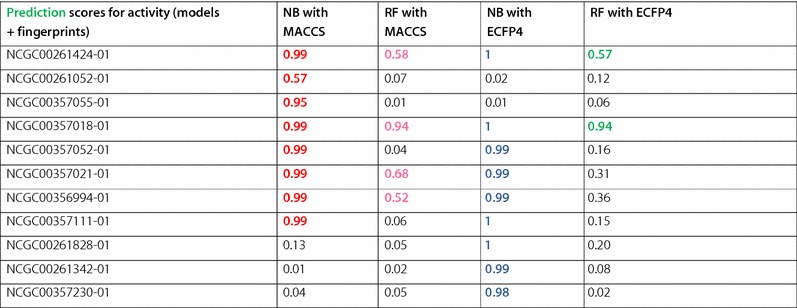
ER-LBD Active compounds correctly predicted in External set using RF and NB models using MACCS and ECFP4 fingerprints

The values correspond to the prediction scores for a compound to be active

**Fig. 4:**
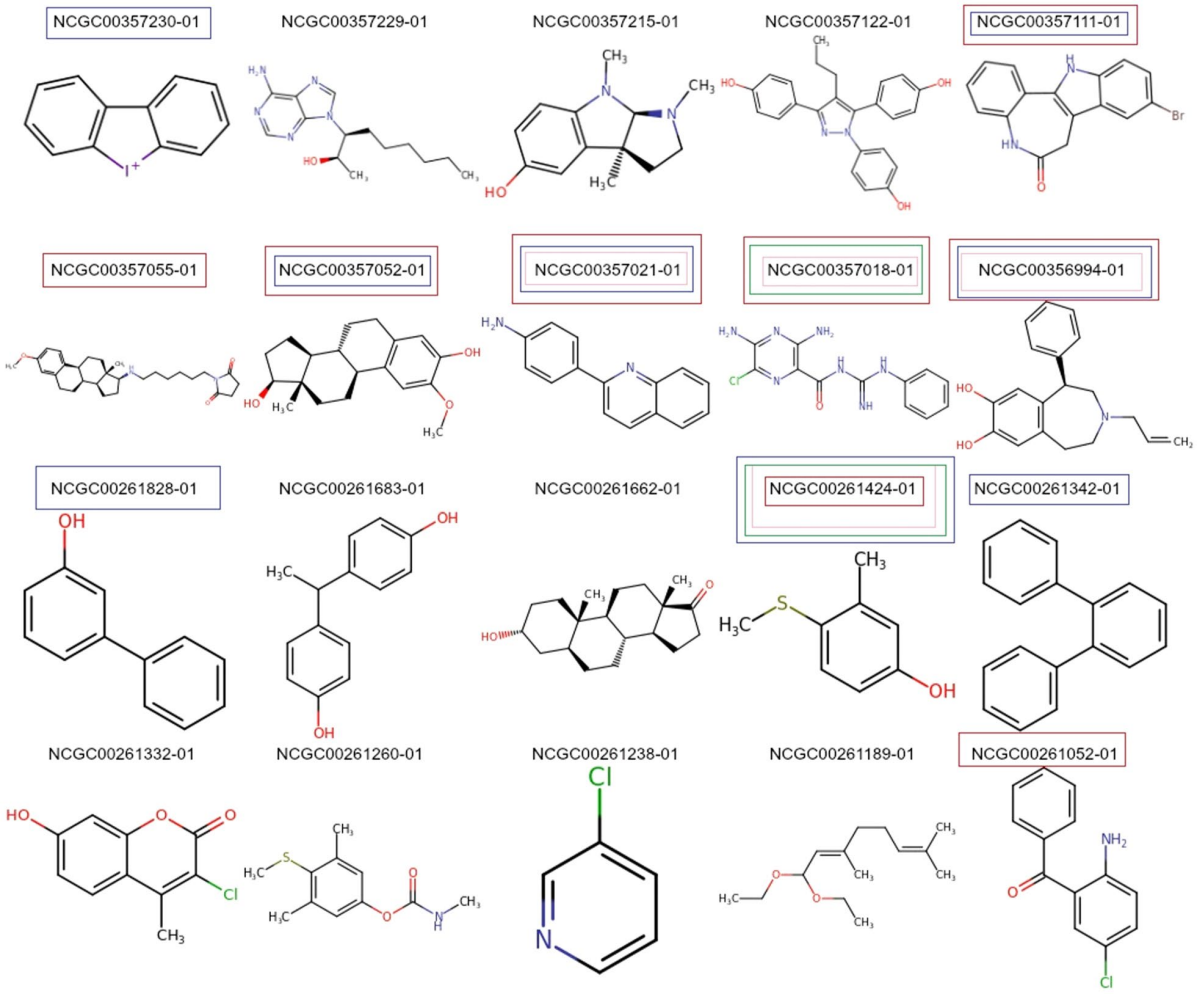
Analysis of chemical space used by descriptors for classification of actives in external sets for ER-LBD target. The above figure shows the different actives present in the external set of ER-LBD. The compounds highlighted in *pink* (MACCS), *green* (ECFP4) are predicted by RF model and *blue* (ECFP4), *red* (MACCS) are predicted by NB models. The respective prediction scores for each classifier are shown in Table 2

Colour denotes different molecules illustrated in the Fig. 4

All the active chemical structures predicted by the RF model were also correctly predicted by the NB model as illustrated in Fig. 4. Additionally, the NB model predicted five additional active compounds correctly whereas the PNN model failed to predict a single active compound. Furthermore, most of the actives in the ER-LBD were correctly predicted by both MACCS and ECFP fingerprints if the functional groups (chloride, bromide, and alcohol) were present in the structures and were found in ‘ortho’ or ‘meta’ position of the ring. On the other hand, the number of false positives in NB models was the highest with 80 incorrect predictions, followed by RF with 4. PNN based models predicted all the inactive structures correctly supporting the fact that the model is biased towards majority class coverage (Table 1).

Additionally, it was observed that the NB based model with both ECFP4 and MACCS fingerprints predicted the active compounds with higher prediction scores compared to RF models (Table 2). It could be because RF fails to predict the active class when the molecules become more complex irrespective of the fingerprints considered (Fig. 4).

### Comparison with Tox21 challenge winners

Finally, we compared the prediction values of the best performing models for all the three targets with those from our previous work [28] and the winning teams from the Tox21 data challenge [29]. Our best performing model, based on RF using MACCS fingerprints, showed slightly better performance than our previous work [28] and performed equally well compared to the challenge winner team for each of the three targets. Furthermore, our combined relatively simple model based on *5*NN and RF using MACCS fingerprints showed, to a small degree, better performance than the Tox21 challenge winner for ER-LBD (Fig. 3).

## Discussion

In the current study, we present a comprehensive comparison of different similarity-based and machine learning methods in predicting the interference of chemical compounds in two major groups of biological pathways, the nuclear receptor pathway and stress response pathway, using the Tox21 screening data. The data, being generated in an uniform experimental setup, provided a gold standard for evaluating performance of different prediction methods.

**Fig. 5:**
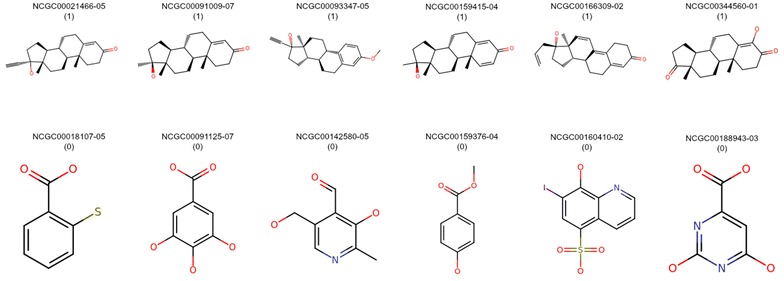
Two-dimensional structures of actives and inactives in the training set for ER-LBD target. A set of training set compounds which are active (1) and inactive (0) against ER-LBD

We noticed that the similarity-weighted *k*NN methods did not perform equally well compared to other machine-learning models for all three targets investigated in this study. A major limitation of the *k*NN approach implemented in this study, although being simple, is that the prediction score for every external set compound heavily depends on the number and diversity of structurally similar active and inactive molecules in the training set, which indirectly determines the number of active and inactive molecules within the *k* neighbours considered. The degree of similarity also plays a key role in deciding which compounds rank among the top *k* neighbours. The average similarity values (Tables 3, 4) of the training set molecules towards individual subsets of actives and inactives of the training set, using three different fingerprints, suggest that the evaluation set compounds are more similar to inactives rather than actives within the training set, explaining the inferior performance of these methods when used individually. It is also widely acknowledged that the “similar-property principle” has exceptions (e.g. activity cliffs) [36, 37]. However, examining the chemical structures of the ER-LBD training set revealed that several compounds consistently have similar molecular frameworks, suggesting that similarity-based approaches play a key role in improving prediction rates, however fail to identify a rare event. The two-dimensional structures of some active molecules containing similar core structures and inactive molecules that are structurally distinct from the former are shown in Fig. 5. This also explains the improvement in performance associated with the ensemble model.

**Table 3:** Average similarity values of external set molecules towards active and inactive subsets of training set for ER-LBD

Fingerprint	Average T against actives	Average T against inactives
MACCS	0.59	0.82
ECFP4	0.29	0.56
ESTATE	0.7	0.91

**Table 4:** Average similarity values of external set molecules (only actives) towards active and inactive subsets of training set for ER-LBD

Fingerprint	Average T against actives	Average T against inactives
MACCS	0.71	0.79
ECFP4	0.41	0.5
ESTATE	0.78	0.94

Moreover, we observed that the RF model is the most accurate classifier producing the most precise results for all three targets. The superior performance of RF models can be attributed to the tuning parameters chosen for individual targets as well as its ability to predict rare events. On the other hand, the inferior performance of PNN models can be explained by its strong inclination towards the majority class (inactive) of the training dataset. Analysing the prediction results revealed that PNN models were able to correctly predict all the negatives in the external validation with a prediction score higher than 0.9 but failed to correctly predict any of the true positives for any target. NB models predicted the highest number of true positives, with prediction scores higher than 0.99, compared to other two methods but the true negative rate was low. However, RF models incorrectly predicted only 4 negatives. This shows that RF models are able to identify the patterns important for the preferred class even when there is a large imbalance in the class distribution within training dataset. It should be noted that the external validation set is also highly imbalanced (Additional file 1: Table S2).

Additionally, it is observed that ToxPrint and Estate fingerprints do not show superior performance compared to standards MACCS and ECFP4 when used with different methods. This could be due to the fact that compounds specific to the targets and assays as such do not have any associated toxicity related alert. However, the presence of substructure patterns in compounds specific to their individual target is more important to predict their activity. Therefore, MACCS fingerprint performed better and consistent with both machine learning and similarity-based approaches. This further adds to the fact that toxicity prediction cannot always be encountered with global approaches such as identification of certain toxic alerts in a chemical compound. Target specificity and local patterns limited to the chemical space used in the study play an important role to predict the activity of new compounds. At the same time, selection of optimal descriptors, which could represent these patterns and an unbiased classifier that can learn the patterns is the essence of a predictive science.

Overall, we emphasize that a simple RF based classifier consistently demonstrated robust prediction for all three targets considered in this study. The prediction accuracies achieved with our best performing machine-learning models were better for all the targets when compared to results based on the RF/ADTree classifier in a recent study performed on the same Tox21 dataset [38]. Furthermore, an ensemble approach that integrates a similarity-weighted *k*NN method with an RF based classifier boosted the performance in case of ER-LBD with an AUC value of 0.83, slightly better than the winning team of the Tox21 Data Challenge [27]. In general, an ensemble model can be effective when an incorrect prediction by one of the individual methods can be compensated by taking into account the prediction of other models [39, 40]. It was also observed in our previous study [28] that predictions obtained using an ensemble model that combines predictions from multiple methods improved the overall prediction.

Finally, the computational costs associated with the training of our best models were very low compared to the Tox21 challenge winning models based on deep learning techniques [41]. This further adds to the usability of our simple yet optimised methods.

## Conclusions

In this study, we emphasize the importance of in silico toxicology as a fast and reliable alternative to reduce the number of animal studies required for evaluation of toxic effects of the ever-increasing new chemical structures. We evaluated different chemical similarity and machine-learning methods using four different types of structural fingerprints as well as molecular descriptors for their performance in predicting the activity of chemicals made available via the Tox21 Data Challenge 2014. The challenge provided a platform for researchers from both academia and industry to evaluate and establish their toxicity/activity prediction models.

Our results suggest that a hybrid strategy that combines similarity-based and machine-learning based prediction models can improve the accuracies of prediction for one of the investigated targets. However, in general, the machine-learning model based on the Random Forest classifier showed the most robust performance. Furthermore, our prediction models were highly consistent with the best-ranked methods from the data challenge and performed better than all the top ten models for ER-LBD.

The findings of our study complement the theme of 3Rs, providing promising and time-saving alternatives to animal trials in evaluating different toxicological endpoints for newly synthesized chemical structures.

## Methods

### Compound datasets, fingerprints and molecular descriptors

The Tox21 10K library is a collection of environmental chemicals and approved drugs with potential to disrupt biological pathways resulting in toxic effects. The chemical structures were directly downloaded from the Tox21 challenge website in structural data format (SDF). The data has now been made freely available on PubChem by the challenge organizers. The complete training sets consist of approximately 10,000 compounds (the total number of molecules varies for different targets) and an external validation set contains 647 chemical structures. Both datasets were standardized using a pipeline explained in our previous work [28]. The steps involved in standardization are removal of water and salts, aromatization, neutralization of charges and addition of explicit hydrogens. Four different types of fingerprints, namely 166-bit MACCS [30], ECFP4 [31], ESTATE [35] and ToxPrint [32–34], and 13 molecular property-based descriptors using RDKit descriptors calculation node in KNIME (Additional file 1: Table S9) were used in our methods. While MACCS, ECFP4 and ESTATE fingerprints and descriptors were calculated using RDKit [42] nodes in KNIME v.2.12.0 [43, 44], ToxPrint fingerprints were generated using the ChemoTyper software version 1.0 [45].

### Similarity search

Three different similarity-weighted *k*NN searches were performed [46] i.e., *3*NN, *5*NN and *7*NN, employing all four types of fingerprints. The Tanimoto coefficient (T) [47] was calculated as the similarity measure. In *k*NN calculations, each evaluation set compound is compared to all training set compounds and the top *k* compounds with highest T values were selected as the nearest neighbours (NNs). The final score was calculated based on the types of the NNs (active or inactive), to arrive at the prediction score for each evaluation set compound.

In particular, if all NNs are either active or inactive, the score was calculated as *score*1 or *score*2, respectively.

$$score1 = \frac{{\mathop \sum \nolimits_{n = 1}^{k} T_{n} }}{k}, \quad score2 = 1 - score1$$

where *k* is the total number of NNs.

Otherwise, the final score is calculated as follows:

$$score3 = \frac{{\mathop \sum \nolimits_{n = 1}^{{k_{a} }} T_{n} }}{{k_{a} }} + \left( {1 - \frac{{\mathop \sum \nolimits_{m = 1}^{{k_{in} }} T_{m} }}{{k_{in} }}} \right)$$

where *k*_*a*_ is the number of active molecules (n) and *k*_*in*_ is the number of inactive molecules (m) among the NNs. All the *k*NN-based predictions, including the cross-validations, were implemented using existing KNIME nodes (Additional file 1: Figures S1, S2) and an additional Java program.

### Machine learning

There are multiple algorithms, which have been used in the field of predictive modeling. Nevertheless we attempted three most popular classification algorithms used in machine learning approaches; NB [48], RF [49] and PNN [50] as shown in Fig. 1. All three classifiers have been previously determined as efficient in terms of classification accuracies as well as computational time [51–53].

#### Naïve Bayes

The NB classifier is based on assumption of the Bayesian theorem of conditional probability, that is for a given target value, the description of each predictor is independent of the other predictions. This method takes into account all descriptor-based properties for the final prediction [48]. This classifier was implemented using the existing NB Learner and Predictor nodes in KNIME (Additional file 1: Figure S3). The maximum number of unique nominal values per attribute was set as 20. The predictor node takes the NB model, test data as input, and as output classifies the test data with an individual prediction score and predicted class.

#### Random Forest

The Random Forest classification is based on decision trees, where each tree is independently constructed and each node is split using the best among the subset of predictors (i.e. individual trees) randomly chosen at the node. RF based model was implemented using the Tree Ensemble Learner and Predictor nodes in KNIME (Additional file 1: Figure S4), which is similar to the RF classifier [49]. The split criterion Gini is used, which has been proven to be a good choice as explained previously [49] and gave the maximum predictive performance for AhR. On the other hand, for ER-LBD and HSE information gain ratio was the optimal split criterion. The number of models (trees) was limited to 1000 and a data sample of 0.8 for AhR and 0.7 for both ER-LBD and HSE was chosen with replacement for each tree; this is similar to bootstrapping. Additionally, a square root function was used for attribute sampling and different sets of attributes were chosen for all the trees. The Predictor node predicts the activity of the test data based on a majority vote in a tree ensemble model with an overall prediction score and individual prediction scores for each class.

### Probabilistic neural network

A PNN is based on a statistical algorithm known as kernel discriminant analysis [54]. PNN operates via a multi-layered feed forward network with four layers. The input layer or the first layer consists of sets of measurements. The pattern layer or the second layer consists of the Gaussian function which uses the given set of data points as centres. The summation layer or the third layer performs an average operation of the outputs from the second layer for each class. The output layer or the fourth layer predicts the class based on votes from largest value [50, 54–56]. PNN based model was implemented with the PNN learner and predictor nodes in KNIME (Additional file 1: Figure S5). All the parameters were kept as default except the maximum number of Epochs was set to 42 to reduce the computational time complexity. The learner node takes numerical data as input and via predictor node the test data is predicted with a score and class.

### Construction of models

A 13-fold cross-validation was performed on the training dataset as described earlier [28] to generate test sets with size similar to the external validation set provided by the Tox21 challenge organizers. This independent set contained 647 chemical structures was used as a second validation set over which the performance (external AUC) of the trained models was evaluated. Four kinds of molecular fingerprints and 13 selected physicochemical descriptors (see Additional file 1: Table S9) were used to represent chemical structures. It was observed that the Tox21 dataset is highly imbalanced with respect to active (minority) and inactive (majority) classes. Detailed statistics on the number of active and inactive molecules for each target are provided in Additional file 1: Tables S1 and S2. Since it was not feasible to enrich the minority class with more compounds for any target, we employed stratified sampling technique during data partitioning to handle this imbalance. Therefore, it was ensured that in each cross-validation run, the ratio of number of active molecules to number of inactive molecules in the test set is similar to that in the training set. Cross-validation [57] was implemented using a meta-node in KNIME that divides training dataset via stratified sampling. A schematic representation of the study methodology is presented in Fig. 1.

### Performance evaluation

A receiver operating characteristic (ROC) curve [58–60], that plots the true positive rate against the false positive rate, was generated to evaluate every model on both cross-validation and external validation test sets. The AUC value was used as a measure to compare the performance of a model with that of other models. The AUC values were calculated using ROC Curve node in KNIME.

## Additional file

10.1186/s13321-016-0162-2 Additional information on the data set and performance of different models and descriptors used in the study. This file contains information on the distribution of training set and external set molecules among active and inactive classes, cross-validation and external validation results for all the models implemented in this study and description of molecular property based descriptors used in this study. The file also contains the methodology and results of SVM approach.

## Abbreviations

AhR: aryl hydrocarbon receptor
AUC: area under the curve
ER-LBD: estrogen receptor ligand binding domain
HSE: heat-shock element
NB: Naїve Bayes classifier
NN: nearest neighbor
PNN: probabilistic neural network
QSAR: quantitative structure–activity relationship
RF: random forest
ROC: receiver operating characteristic
T: Tanimoto coefficient
Tox21: toxicology in the 21st century
