# Supplementary material for: Computational methods for prediction of in vitro effects of new chemical structures
Source: J Cheminform. 2016 Sep 29;8:51. doi: 10.1186/s13321-016-0162-2 (PMC5043617; doi:10.1186/s13321-016-0162-2)
Supplement: Supplementary file 1 — 10.1186/s13321-016-0162-2 Additional information on the data set and performance of different models and descriptors used in the study. This file contains information on the distribution of training set and external set molecules among active and inactive classes, cross-validation and external validation results for all the models implemented in this study and description of molecular property based descriptors used in this study. The file also contains the methodology and results of SVM approach. [file 13321_2016_162_MOESM1_ESM.docx]

Additional information on the data set and performance of different models and descriptors used in the study

# **Computational methods for prediction of *in vitro* effects of new chemical structures**

Priyanka Banerjee^1,3,†^, Vishal B. Siramshetty^2,4,†^, Malgorzata N. Drwal^1,§,*^, Robert Preissner^1,2,4^

^1^Structural Bioinformatics Group, Institute for Physiology, Charité – University Medicine Berlin, Berlin, Germany

^2^Structural Bioinformatics Group, Experimental and Clinical Research Center (ECRC), Charité – University Medicine Berlin, Berlin, Germany

^3^Graduate School of Computational Systems Biology, Humboldt University of Berlin, Berlin, Germany

^4^BB3R – Berlin Brandenburg 3R Graduate School, Free University of Berlin, Berlin, Germany

^†^These are the joint first authors of this work

^§^Corresponding author

^*^Current address: Laboratoire d'innovation thérapeutique, Université de Strasbourg, Illkirch, France

Email addresses:

PB: priyanka.banerjee@charite.de

VB: vishal.siramshetty@charite.de

MND: [malgorzata.drwal@alumni.charite.de](mailto:malgorzata.drwal@alumni.charite.de)

RP: [robert.preissner@charite.de](mailto:robert.preissner@charite.de)

**Table S1 - Training set class distribution.**

| **Target** | **Total compounds** | **Number of actives** | **Number of inactives** | **Ratio (active/ inactives)** |
| --- | --- | --- | --- | --- |
| AhR | 6901 | 769 | 6132 | 0.125 |
| ER-LBD | 6801 | 346 | 6455 | 0.053 |
| HSE | 7328 | 308 | 7019 | 0.043 |

**Table S2 - External set class distribution.**

| **Targets** | **Total compounds** | **Number of actives** | **Number of inactives** | **Ratio (active/ inactives)** |
| --- | --- | --- | --- | --- |
| AhR | 610 | 73 | 537 | 0.135 |
| ER-LBD | 600 | 20 | 580 | 0.034 |
| HSE | 610 | 23 | 588 | 0.039 |

##

## **Table S3 - Cross validation results for all top performing models.**

Mean area under the curve (AUC) from receiver-operating characteristic (ROC) analysis.

| **Target** | **AhR** | **ER-LBD** | **HSE** |
| --- | --- | --- | --- |
| ***k*-nearest neighbor (*3*NN)** | 0.82 | 0.80 | 0.76 |
| ***k*-nearest neighbor (*5*NN)** | 0.82 | 0.81 | 0.74 |
| ***k*-nearest neighbor (*7*NN)** | 0.81 | 0.81 | 0.73 |
| **Naive Bayes** | 0.83 | 0.76 | 0.71 |
| **Random Forest** | 0.90 | 0.86 | 0.80 |
| **Probabilistic Neural Network** | 0.84 | 0.78 | 0.71 |
| **Previous method (Drwal et al. 2015)** | 0.90 | 0.86 | 0.83 |
| **Ensemble (*5*NN + RF)** | 0.81 | 0.85 | 0.80 |

## **Table S4 - External validation results for all top performing models.**

Area under the curve (AUC) from receiver-operating characteristic (ROC) analysis.

| **Target** | **AhR** | **ER-LBD** | **HSE** |
| --- | --- | --- | --- |
| ***k*-nearest neighbor (*3*NN)** | 0.80 | 0.70 | 0.80 |
| ***k*-nearest neighbor (*5*NN)** | 0.81 | 0.71 | 0.79 |
| ***k*-nearest neighbor (*7*NN)** | 0.80 | 0.70 | 0.79 |
| **Naive Bayes** | 0.84 | 0.71 | 0.79 |
| **Random Forest** | 0.90 | 0.81 | 0.86 |
| **Probabilistic Neural Network** | 0.85 | 0.77 | 0.77 |
| **Previous method (Drwal et al. 2015)** | 0.89 | 0.79 | 0.85 |
| **Tox21 winner** | 0.92 | 0.82 | 0.86 |
| **Ensemble (*5*NN + RF)** | 0.90 | 0.83 | 0.86 |

**Table S5 - Cross validation results for different *k*NN methods using MACCS, ECFP4, ESTATE and ToxPrint fingerprints.**

Mean area under the curve (AUC) from receiver-operating characteristic (ROC) analysis.

| **Method** | **Fingerprint** | **AhR** | **ER-LBD** | **HSE** |
| --- | --- | --- | --- | --- |
| ***3/5/7-*NN** | **MACCS** | 0.82/ 0.82/ 0.81 | 0.80/ 0.81/ 0.81 | 0.76/ 0.74/ 0.73 |
| ***3/5/7-*NN** | **ECFP4** | 0.57/ 0.47/ 0.41 | 0.62/ 0.51/ 0.44 | 0.63/ 0.5/ 0.42 |
| ***3/5/7-*NN** | **ESTATE** | 0.8/ 0.76/ 0.74 | 0.68/ 0.71/ 0.68 | 0.75/ 0.72/ 0.7 |
| ***3/5/7-*NN** | **ToxPrint** | 0.84/ 0.84/ 0.81 | 0.75/ 0.74/ 0.74 | 0.81/ 0.79/ 0.77 |

**Table S6 - External validation results for different *k*NN methods using MACCS, ECFP4, ESTATE and ToxPrint fingerprints.**

| **Method** | **Fingerprint** | **AhR** | **ER-LBD** | **HSE** |
| --- | --- | --- | --- | --- |
| ***3/5/7-*NN** | **MACCS** | 0.8/ 0.81/ 0.8 | 0.7/ 0.71/ 0.7 | 0.8/ 0.79/ 0.79 |
| ***3/5/7-*NN** | **ECFP4** | 0.59/ 0.55/ 0.52 | 0.52/ 0.45/ 0.43 | 0.6/ 0.57/ 0.5 |
| ***3/5/7-*NN** | **ESTATE** | 0.72/ 0.78/ 0.74 | 0.48/ 0.54/ 0.57 | 0.73/ 0.74/ 0.7 |
| ***3/5/7-*NN** | **ToxPrint** | 0.77/ 0.76/ 0.76 | 0.63/ 0.6/ 0.62 | 0.65/ 0.64/ 0.66 |

**Table S7 - Cross validation results for RF, NB PNN and SVM models with fingerprints alone and fingerprints in combination with different molecular descriptors.**

Mean area under the curve (AUC) from receiver-operating characteristic (ROC) analysis.

| **Model** | **Features** | **AhR** | **ER-LBD** | **HSE** |
| --- | --- | --- | --- | --- |
| **RF** | **MACCS** | 0.90 | 0.83 | 0.78 |
| **RF** | **MACCS + descriptors** | 0.91 | 0.86 | 0.80 |
| **RF** | **Estate** | 0.77 | 0.59 | 0.66 |
| **RF** | **Estate + descriptors** | 0.79 | 0.55 | 0.67 |
| **RF** | **ECFP4** | 0.87 | 0.82 | 0.77 |
| **RF** | **ECFP4 + descriptors** | 0.89 | 0.83 | 0.79 |
| **RF** | **ToxPrint** | 0.88 | 0.80 | 0.78 |
| **RF** | **ToxPrint + descriptors** | 0.88 | 0.85 | 0.80 |
| **NB** | **MACCS** | 0.83 | 0.73 | 0.67 |
| **NB** | **MACCS + descriptors** | 0.82 | 0.73 | 0.63 |
| **NB** | **Estate** | 0.81 | 0.73 | 0.71 |
| **NB** | **Estate + descriptors** | 0.79 | 0.73 | 0.63 |
| **NB** | **ECFP4** | 0.77 | 0.76 | 0.70 |
| **NB** | **ECFP4 + descriptors** | 0.78 | 0.77 | 0.69 |
| **NB** | **ToxPrint** | 0.83 | 0.71 | 0.68 |
| **NB** | **ToxPrint + descriptors** | 0.80 | 0.72 | 0.70 |
| **PNN** | **MACCS** | 0.84 | 0.78 | 0.71 |
| **PNN** | **MACCS + descriptors** | 0.82 | 0.79 | 0.72 |
| **PNN** | **Estate** | 0.83 | 0.77 | 0.69 |
| **PNN** | **Estate + descriptors** | 0.82 | 0.78 | 0.70 |
| **PNN** | **ECFP4** | 0.84 | 0.77 | 0.71 |
| **PNN** | **ECFP4 + descriptors** | 0.79 | 0.77 | 0.68 |
| **PNN** | **ToxPrint** | 0.83 | 0.76 | 0.68 |
| **PNN** | **ToxPrint + descriptors** | 0.82 | 0.78 | 0.67 |
| **SVM** | **MACCS** | 0.80 | 0.50 | 0.52 |
| **SVM** | **MACCS + descriptors** | 0.78 | 0.59 | 0.63 |
| **SVM** | **Estate** | 0.66 | 0.50 | 0.45 |
| **SVM** | **Estate + descriptors** | 0.52 | 0.51 | 0.49 |
| **SVM** | **ECFP4** | 0.76 | 0.76 | 0.69 |
| **SVM** | **ECFP4 + descriptors** | 0.77 | 0.74 | 0.68 |
| **SVM** | **ToxPrint** | 0.80 | 0.76 | 0.73 |
| **SVM** | **ToxPrint + descriptors** | 0.80 | 0.73 | 0.75 |

**Table S8 - External validation results RF, NB PNN and SVM models with fingerprints alone and fingerprints in combination with different molecular descriptors.**

Area under the curve (AUC) from receiver-operating characteristic (ROC) analysis.

| **Model** | **Features** | **AhR** | **ER-LBD** | **HSE** |
| --- | --- | --- | --- | --- |
| **RF** | **MACCS** | 0.90 | 0.81 | 0.86 |
| **RF** | **MACCS + descriptors** | 0.87 | 0.77 | 0.81 |
| **RF** | **Estate** | 0.78 | 0.51 | 0.87 |
| **RF** | **Estate + descriptors** | 0.76 | 0.74 | 0.73 |
| **RF** | **ECFP4** | 0.87 | 0.72 | 0.81 |
| **RF** | **ECFP4 + descriptors** | 0.88 | 0.78 | 0.83 |
| **RF** | **ToxPrint** | 0.89 | 0.71 | 0.70 |
| **RF** | **ToxPrint + descriptors** | 0.74 | 0.62 | 0.69 |
| **NB** | **MACCS** | 0.82 | 0.69 | 0.79 |
| **NB** | **MACCS + descriptors** | 0.84 | 0.70 | 0.77 |
| **NB** | **Estate** | 0.79 | 0.67 | 0.72 |
| **NB** | **Estate + descriptors** | 0.78 | 0.67 | 0.60 |
| **NB** | **ECFP4** | 0.77 | 0.71 | 0.76 |
| **NB** | **ECFP4 + descriptors** | 0.78 | 0.71 | 0.74 |
| **NB** | **ToxPrint** | 0.82 | 0.63 | 0.63 |
| **NB** | **ToxPrint + descriptors** | 0.83 | 0.60 | 0.60 |
| **PNN** | **MACCS** | 0.81 | 0.69 | 0.77 |
| **PNN** | **MACCS + descriptors** | 0.82 | 0.76 | 0.72 |
| **PNN** | **Estate** | 0.78 | 0.68 | 0.76 |
| **PNN** | **Estate + descriptors** | 0.84 | 0.77 | 0.53 |
| **PNN** | **ECFP4** | 0.85 | 0.69 | 0.70 |
| **PNN** | **ECFP4 + descriptors** | 0.82 | 0.75 | 0.67 |
| **PNN** | **ToxPrint** | 0.82 | 0.69 | 0.67 |
| **SVM** | **MACCS** | 0.84 | 0.72 | 0.71 |
| **SVM** | **MACCS + descriptors** | 0.83 | 0.74 | 0.75 |
| **SVM** | **Estate** | 0.82 | 0.50 | 0.55 |
| **SVM** | **Estate + descriptors** | 0.74 | 0.70 | 0.59 |
| **SVM** | **ECFP4** | 0.75 | 0.76 | 0.64 |
| **SVM** | **ECFP4 + descriptors** | 0.76 | 0.70 | 0.61 |
| **SVM** | **ToxPrint** | 0.83 | 0.74 | 0.70 |
| **SVM** | **ToxPrint + descriptors** | 0.85 | 0.79 | 0.71 |

**Support vector machine (Methodology):**

The support vector machine (SVM) based model was implemented using the existing KNIME node (LIBSVM v2.89) [1]. Two different kernel functions linear and polynomial, were evaluated by performing cross-validation. The linear function performed better and hence was used for further analysis. The overall performance of the SVM model was poor compared to other models and hence was not included for comparison with top performing models in the main manuscript. Additionally, an SVM model using the radial basis function (rbf) was reported by Koutsokous, et.al (2016) [2], on the same data set. The results reported in our study are better than the performance of the rbf-based SVM model.

**Table S9 - Molecular descriptors used in combination with fingerprints.**

| **Descriptor name** | **Description** |
| --- | --- |
| SlogP | Log of the octanol/water partition coefficient |
| Chi0v | Atomic valence connectivity index (order 0) |
| Chi1v | Atomic valence connectivity index (order 1) |
| Chi2v | Atomic valence connectivity index (order 2) |
| Chi3v | Atomic valence connectivity index (order 3) |
| Chi4v | Atomic valence connectivity index (order 4) |
| Chi1n | Simple molecular connectivity index for path (order 1) |
| Chi2n | Simple molecular connectivity index for path (order 2) |
| Chi3n | Simple molecular connectivity index for path (order 3) |
| Chi4n | Simple molecular connectivity index for path (order 4) |
| kappa1 | Kappa index for 1 bonded fragment |
| kappa1 | Kappa index for 2 bonded fragment |
| kappa1 | Kappa index for 3 bonded fragment |

Based on statistical and literature analysis, a total of 43 molecular property based descriptors were calculated for all the molecules in training set and test set using the RDKit descriptor calculation node in KNIME. We examined the original derivation and significance of each descriptor in the literature, and eliminated those descriptors with primary function not involved in toxicity and ligand-receptors interactions. All the remaining descriptor values were normalized using Z score normalization in KNIME. All the descriptors with missing values were removed. Additionally, descriptors with low variance to both active and inactive classes were removed. The final list of 13 descriptors used in this study is presented above. The SlogP descriptor accounts to hydrophobic and hydrophilic effect [3, 4]. Chi indices takes into consideration the valence value to encode sigma, pi and lone pair introduction [5]. Kappa shape descriptors compare the molecule with extreme shape for that number of atoms [6]. The Kappa shape descriptors have been widely used in the field of *in silico* toxicity prediction [7–10].


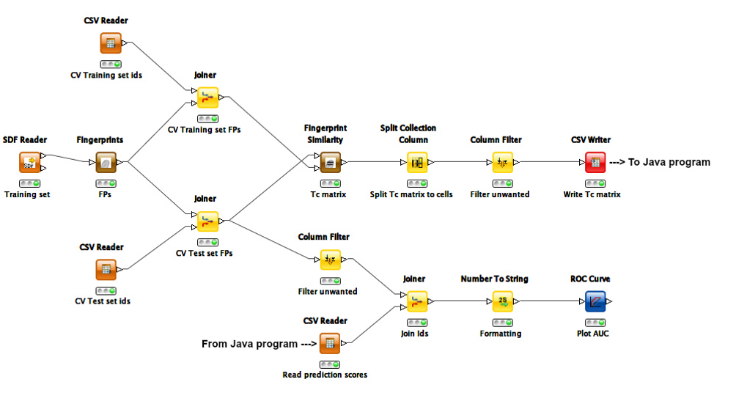


**Figure S1: Cross validation performed for similarity search based model using KNIME workflow 1.**


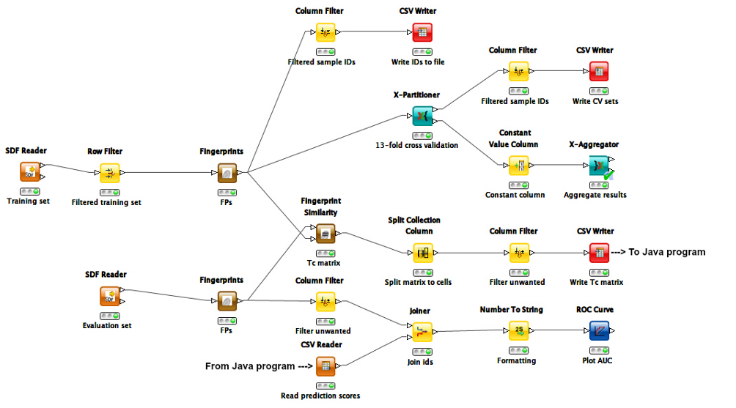
 **Figure S2: Cross validation performed for similarity search based model using KNIME workflow 2.**
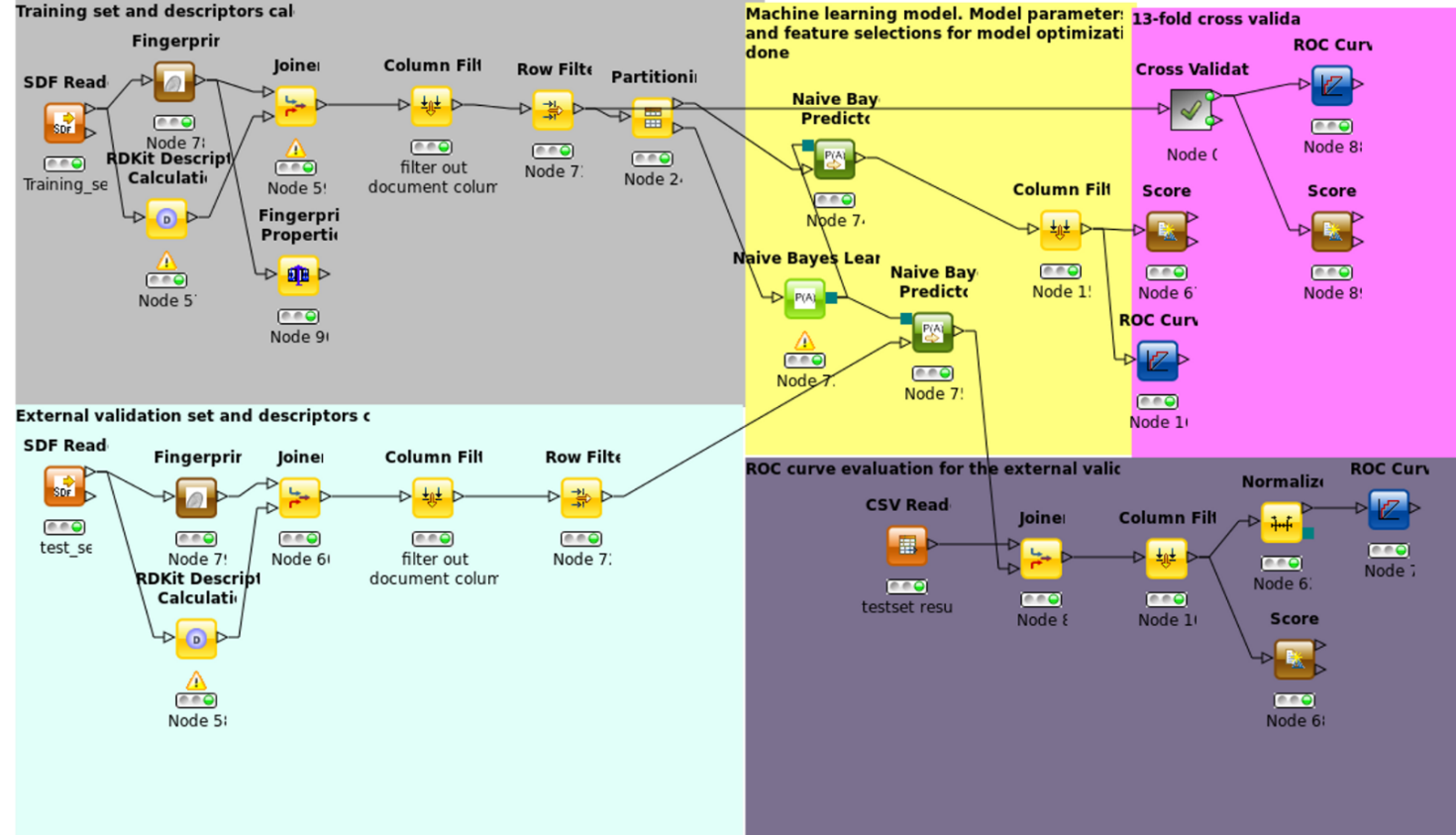


**Figure S3: KNIME workflow for Naïve Bayes classifier**


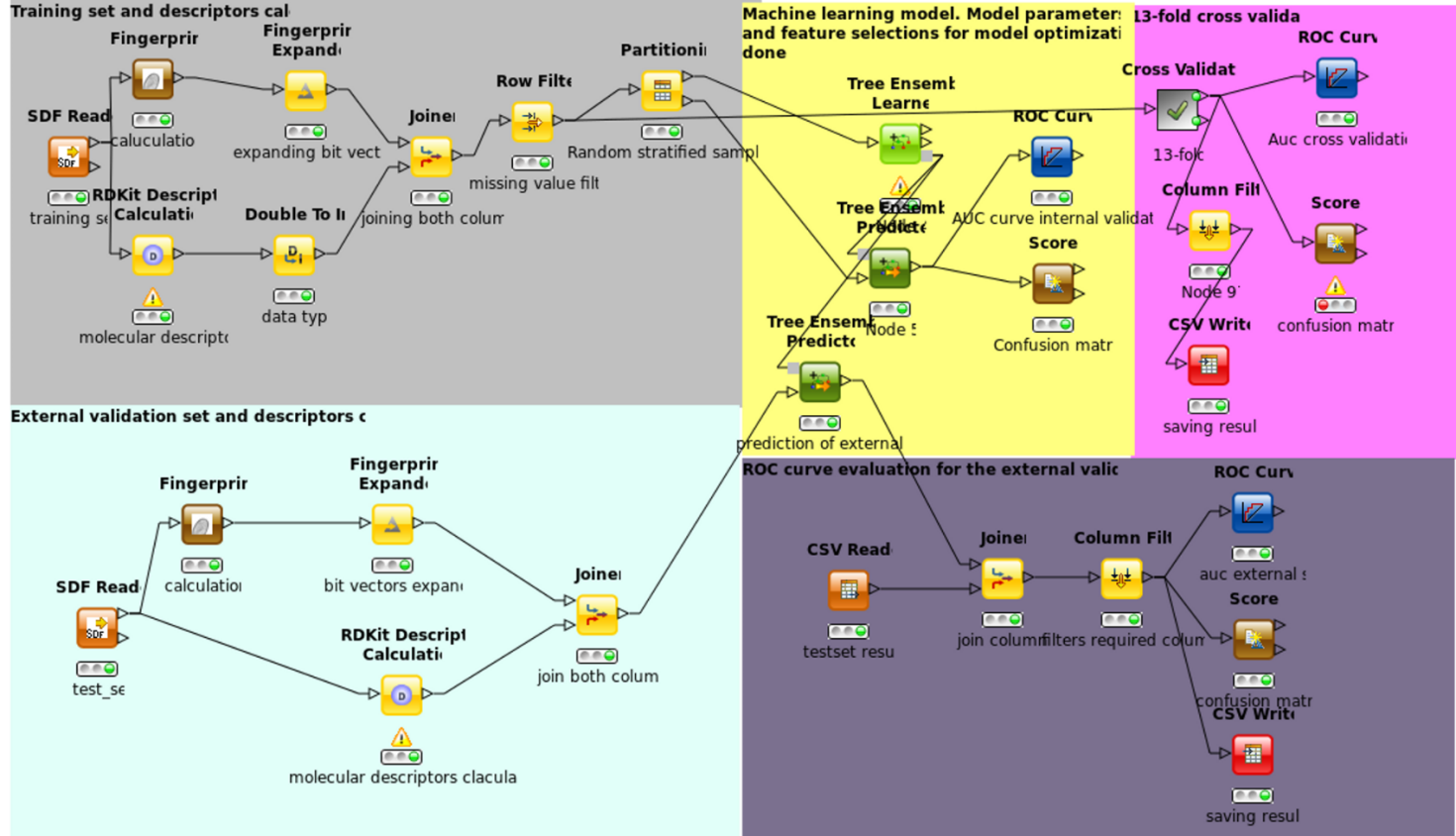


**Figure S4: KNIME workflow for Random Forest classifier**


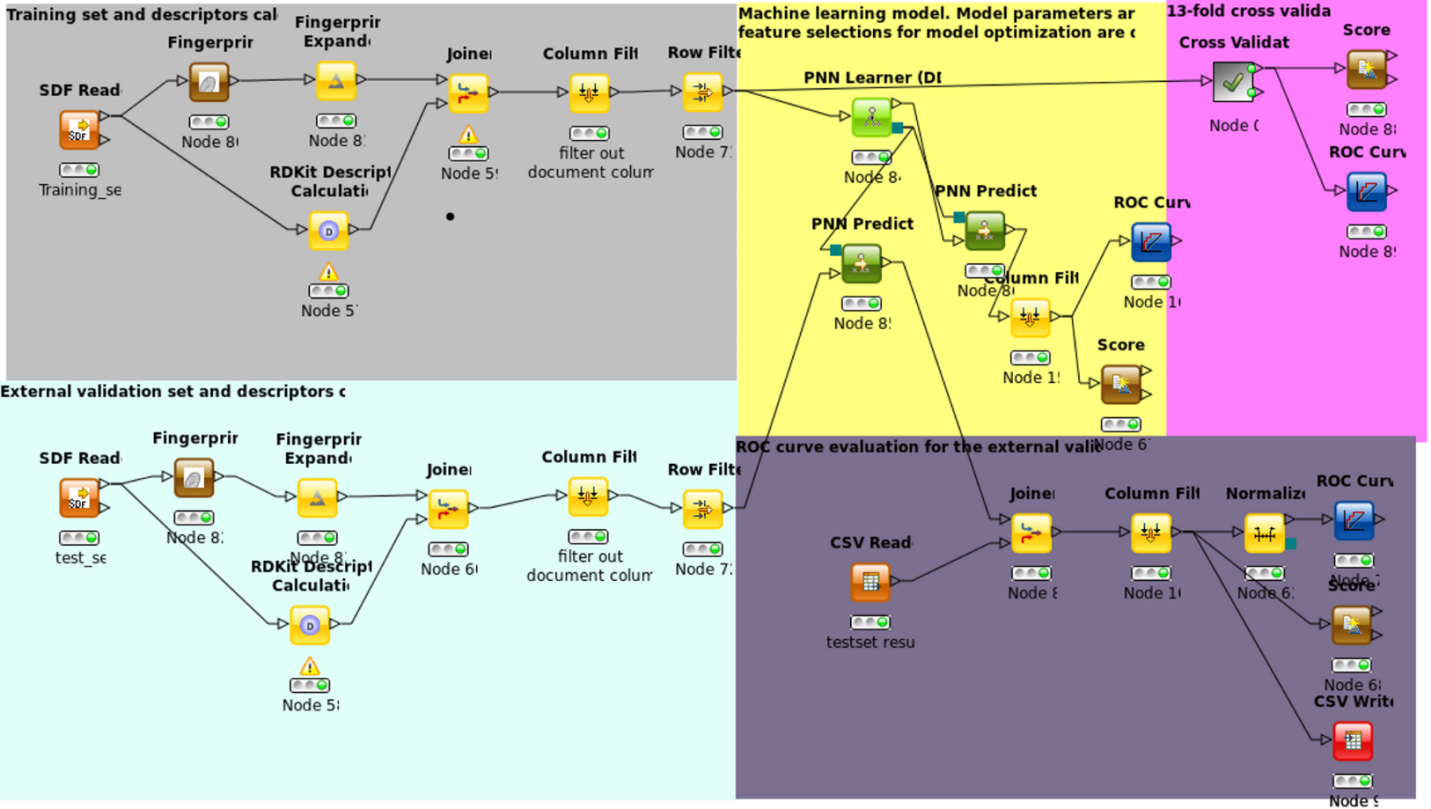
**Figure S5: KNIME workflow for Probabilistic Neural Network classifier**

**List of abbreviations**

AhR - aryl hydrocarbon receptor; AUC - area under the curve; ER-LBD - estrogen receptor ligand binding domain; HSE - heat-shock element; NB - Naїve Bayes classifier; NN - nearest neighbor; PNN - probabilistic neural network; QSAR - quantitative structure-activity relationship; RF - random forest; ROC - receiver operating characteristic; T_c_ or Tc - Tanimoto coefficient; Tox21 - Toxicology in the 21st century

**References**

1. Chang C, Lin C: **LIBSVM : A Library for Support Vector Machines**. 2011, **2**.

2. Koutsoukas A, Amand JS, Mishra M, Huan J: **Predictive Toxicology : Modeling Chemical Induced Toxicological Response Combining Circular Fingerprints with Random Forest and Support Vector Machine**. 2016, **4**(March):1–9.

3. Hou TJ, Xu XJ: **ADME Evaluation in Drug Discovery. 3. Modeling Blood-Brain Barrier Partitioning Using Simple Molecular Descriptors**. *Journal of Chemical Information and Computer Sciences* 2003, **43**:2137–2152.

4. Todeschini R, Consonni V: *Molecular Descriptors for Chemoinformatics*. 2010.

5. Todeschini R, Consonni V: **Handbook of Molecular Descriptors**. *New York* 2000, **11**:688.

6. Hall LH, Kier LB: **The Molecular Connectivity Chi Indexes and Kappa Shape Indexes in Structure-Property Modeling**. In *Reviews in Computational Chemistry*. *Volume 2*; 2007:367–422.

7. Trohalaki S, Pachter R, Geiss KT, Frazier JM: **Halogenated aliphatic toxicity QSARs employing metabolite descriptors.** *Journal of chemical information and computer sciences* 2004, **44**:1186–92.

8. Llewellyn LE: **Predictive toxinology: An initial foray using calculated molecular descriptors to describe toxicity using saxitoxins as a model**. *Toxicon* 2007, **50**:901–913.

9. Arulmozhiraja S, Morita M: **Structure-Activity Relationships for the Toxicity of Polychlorinated Dibenzofurans: Approach through Density Functional Theory-Based Descriptors**. *Chemical Research in Toxicology* 2004, **17**:348–356.

10. Katritzky a R, Tatham DB, Maran U: **Theoretical descriptors for the correlation of aquatic toxicity of environmental pollutants by quantitative structure-toxicity relationships.** *Journal of chemical information and computer sciences* 2001, **41**:1162–76.
